# New-Onset Atrial Fibrillation Following Isolated Coronary Artery Bypass Grafting: Is Pulmonary Hypertension a Risk Factor?

**DOI:** 10.21470/1678-9741-2024-0352

**Published:** 2025-08-22

**Authors:** Barış Akça, Nevzat Erdil

**Affiliations:** 1 Department of Cardiovascular Surgery, Faculty of Medicine, Inonu University, Malatya, Turkey; 2 Department of Cardiovascular Surgery, Ministry of Health Antalya City Hospital, Antalya, Turkey

**Keywords:** Atrial Fibrillation, Coronary Artery Bypass, Risk Factors, Hypertension, Pulmonary.

## Abstract

**Introduction:**

This study aimed to clarify whether pulmonary hypertension is a risk factor for postoperative new-onset atrial fibrillation (NOAF) following isolated coronary artery bypass grafting (CABG).

**Methods:**

Data of 4,782 patients were retrospectively examined from clinical database, and data of isolated CABG performed patients (n = 854) with preoperative echocardiography including pulmonary artery pressure (PAP) measurement were enrolled in study. While 115 patients had post-CABG NOAF (atrial fibrillation [AF] group), 739 did not have AF (non-AF group). Demographic, clinical, and treatment-related parameters were compared between groups, and independent clinical predictors of NOAF were identified by multivariate analysis.

**Results:**

Patients of AF group were significantly older and had higher European System for Cardiac Operative Risk Evaluation (EuroSCORE) points, significantly elevated mean systolic PAP, and more pulmonary hypertension. Multivariate regression analysis revealed that mean systolic PAP (odds ratio [OR]: 1.027, 95% confidence interval [CI]: 1.006 - 1.048) and pulmonary hypertension (≥ 30 mmHg; OR: 1.659, 95% CI: 1.093 - 2.518) were independent risk factors for post-CABG NOAF. Chronic obstructive pulmonary disease (COPD) (OR: 2.033, 95% CI: 1.265 - 3.268) and mean duration of ventilation support (OR: 1.059, 95% CI: 1.017 - 1.104) were additionally determined as risk factors for post-CABG NOAF.

**Conclusion:**

This study identified patients’ age, high EuroSCORE points, presence of COPD, prolonged ventilation support, and increased PAP as predictors of post-CABG NOAF. Understanding the risk factors will provide better guidance in preventing this complication and its potential consequences. Prospective randomized controlled trials are required to further validate these findings and provide more robust evidence.

## INTRODUCTION

**Table:** 

Abbreviations, Acronyms & Symbols				
AF	= Atrial fibrillation		ICU	= Intensive care unit
BMI	= Body mass index		LITA	= Left internal thoracic artery
BSA	= Body surface area		LMCA	= Left main coronary artery
CABG	= Coronary artery bypass grafting		LVEF	= Left ventricular ejection fraction
CAD	= Coronary artery disease		NOAF	= New-onset atrial fibrillation
CI	= Confidence interval		OR	= Odds ratio
COPD	= Chronic obstructive pulmonary disease		PAP	= Pulmonary artery pressure
CPB	= Cardiopulmonary bypass		PH	= Pulmonary hypertension
ECG	= Electrocardiogram		PTCA	= Percutaneous transluminal coronary angioplasty
EuroSCORE	= European System for Cardiac Operative Risk Evaluation		RCA	= Right coronary artery
IABP	= Intra-aortic balloon pump		TTE	= Transthoracic echocardiography

The most prevalent arrhythmia after coronary artery bypass grafting (CABG) is new-onset atrial fibrillation (NOAF); its incidence ranges from 15% to 50% and it is associated with high mortality and morbidity rates and longer hospital stays^[1-4]^. Despite advancements in surgical techniques and pre and perioperative care, identifying the risk factors for NOAF remains crucial for patients who undergo cardiac surgery to optimize patient management strategies and improve postoperative outcomes.

The leading cause of pulmonary hypertension (PH) is left-sided heart disease^[5]^. One of the primary factors contributing to higher rates of mortality and morbidity following open heart surgery is PH. Its association with adverse cardiovascular outcomes has been studied and the development of atrial arrhythmia in patients with PH was shown to be related to poor prognosis^[6,7]^.

In light of previous studies that have provided evidence suggesting a relationship between PH and atrial arrythmias, this study aimed to clarify whether PH is a risk factor for the development of post-CABG NOAF.

## METHODS

The data of 4,782 patients were retrospectively examined from a clinical database from March 2002 to December 2022. The patients (n = 854) who had undergone isolated CABG with preoperative echocardiography including pulmonary artery pressure (PAP) measurements were enrolled in the study. These patients were respectively assigned to the atrial fibrillation (AF) group (n = 115) or the non-AF group (n = 739) based on whether or not they developed NOAF following isolated CABG. PH (≥ 30 mmHg) and other preoperative, perioperative, and postoperative demographic, clinical, and disease-related parameters were assessed to determine whether they were risk factors for post-CABG NOAF. Predictive power calculations were performed for all identified risk factors.

Patients with implanted pacemakers, hyperthyroidism or hypothyroidism, accompanying valve pathologies, AF or any other type of rhythm disorder in previous years, post-myocardial infarction ventricular septal defect, additional valvular procedures together with the CABG operation, or revision surgery were excluded from the study. Patients with a single episode of short-lasting postoperative AF were also excluded from the study. The ethics committee of the Faculty of Medicine of the Inonu University approved the study protocol (2024/5600), and the research was conducted in line with the principles of the Declaration of Helsinki.

### Anesthesia

All patients were first monitored on the operating table. Peripheral arterial oxygen saturation was monitored using a pulse oximetry probe, while arterial blood gas and systemic arterial blood pressure were monitored via a 20-G intravenous catheter inserted into the right radial artery. Anesthesia induction was achieved using a combination of medications administered through a peripheral venous catheter, including fentanyl citrate (5 µg/kg), 2% lidocaine hydrochloride (1 mg/kg), vecuronium bromide (0.1 mg/kg), and midazolam hydrochloride (0.2 - 0.3 mg/kg). Following complete muscle relaxation, patients were manually respirated, intubated, and connected to a ventilator. Anesthesia maintenance was achieved using a continuous infusion of fentanyl citrate (10 to 30 µg/kg) and midazolam hydrochloride (0.1 to 0.3 mg/kg/h), adjusted based on individual hemodynamic conditions. Before the surgical incision, all patients received intravenous prophylactic antibiotic therapy in the form of 1 g of cefazolin sodium.

### Surgical Technique

Operations were carried out by median sternotomy with general anesthesia. The choice between on-pump and off-pump CABG techniques was made at the discretion of the surgeon. Among the cohort, 731 patients underwent conventional on-pump CABG utilizing a roller pump system, oxygenator without heparin surface, two-stage venous cannula, and tubing set for cardiopulmonary bypass (CPB). CPB was maintained by non-pulsatile pumping flow in the range of 2.4 - 3.5 L/min/m^2^ and mild systemic hypothermia of 32 - 34°C. Mean arterial pressure and hematocrit level were meticulously regulated during CPB to remain within the ranges of 50 - 70 mmHg and 22-25%, respectively. Anticoagulation was initiated via heparin administration prior to CPB, ensuring an activated clotting time > 450 seconds. Myocardial protection during CPB encompassed both antegrade and retrograde cold blood cardioplegia, with warm blood cardioplegia administered immediately preceding aortic cross-clamp removal. Protamine sulfate was employed to reverse the effects of the heparin after CPB. In contrast, 123 patients (14.4%) underwent off-pump CABG, where anastomosis was performed using stabilization and retractor equipment. A transient epicardial pace (Flexon™ 3-0, Syneture, Covidien, Mansfield, Massachusetts, United States of America) was routinely positioned on the right ventricle for all patients. The internal thoracic artery was the preferred conduit for left anterior descending artery anastomosis, resorting to saphenous vein grafts when internal thoracic artery suitability was compromised. In patients with multivessel coronary artery disease, saphenous vein grafts and/or radial arteries were used alongside internal thoracic artery grafts. Radial artery grafts were selectively employed for vessels exhibiting stenosis > 70%, excluding the left anterior descending artery. In on-pump surgeries, proximal and distal anastomoses were conducted during cross-clamping using 6/0 and 7 - 8/0 polypropylene sutures, respectively. Administration of N-acetyl cysteine, theophylline ethylenediamine, and methylprednisolone preceded cross-clamp removal in all on-pump procedures.

Beta-blockers (metoprolol or nebivolol) were used for all patients both preoperatively and postoperatively with dosages tailored to individual hemodynamic responses during hospitalization. Calcium channel blockers such as diltiazem or nifedipine were the preferred choice postoperatively, particularly in cases in which a radial artery graft was utilized. Nebivolol was often additionally prescribed alongside nifedipine administration. As part of the standard postoperative routine, low-molecular-weight heparin was administered to every patient once a day until discharge. Other routine postoperative medications included acetylsalicylic acid, atorvastatin, and nitrate. Angiotensin receptor blockers were used in cases of postoperative hypertension.

### Data Collection and Definitions

Following a retrospective examination of patient records from the clinical database, 854 patients with full data for the relevant preoperative, perioperative, and postoperative parameters were included in this study. PH and other parameters were compared between the non-AF and AF groups. All included patients had systolic PAP of ≥ 30 mmHg as measured by transthoracic echocardiography (TTE). As in daily routine, the cardiology clinic of the hospital performed TTE for all patients included in this study. TTE was performed with patients in the left lateral decubitus position using parasternal longand short-axis views, as well as the apical four-chamber window. TTE was performed using HDI 5000 CV (ATL, Philips, Bothell, Washington, United States of America) and IE33 (Philips, Unites States of America) devices. The acoustic window providing the best visualization of the tricuspid regurgitation jet in color Doppler echocardiography is one where the angle between the ultrasound beam and the flow direction is minimized, and this was preferred for accurately measuring systolic PAP using the modified Bernoulli equation. An approximate right atrial pressure was then added to this value for precise systolic PAP measurement. In the event of different PAP measurements in alternative echocardiographic windows, the upper value was recorded.

Continuous cardiac rhythm monitoring was performed throughout patients’ intensive care unit (ICU) stays. As part of the daily routine, a 12-lead electrocardiogram (ECG) was obtained for each patient without delay upon admission to the ICU and subsequently on the postoperative first, second, and fourth days, as well as prior to hospital discharge. Moreover, following the move to the in-patient clinic after patients left the ICU, medical assistants and nurses diligently monitored radial pulse rates of all patients four times daily. Concurrently, they conducted assessments for symptoms indicative of arrhythmia during each pulse check. In the event of clinical manifestations suggesting arrhythmia, immediate ECG documentation was obtained for the patient. The doctor’s evaluation of the ECG findings was used to finalize the diagnosis of AF. Patients were identified as having AF in the presence of rapid oscillations on ECG together with fibrillatory P waves characterized by variations in timing, shape, and size or absent P waves accompanied by irregular QRS complexes persisting over five minutes and requiring treatment. Subsequently, patients diagnosed with AF were managed according to a standardized protocol involving anticoagulation therapy and the administration of amiodarone. Furthermore, preoperative renal insufficiency was characterized by a serum creatinine level of ≥ 1.5 mg/dL before CABG^[8,9]^.

### Statistical Analysis

Statistical analysis was carried out with IBM Corp. Released 2016, IBM SPSS Statistics for Windows, version 24.0, Armonk, NY: IBM Corp. The Shapiro-Wilk test was used to evaluate the assumption of normality. Normally distributed continuous variables were reported as mean ± standard deviation and were analyzed with the independent samples *t*-test. The chi-square test or Fisher’s exact test was used in assessing categorical variables. Parameters with a significance level of *P* ≤ 0.20 according to univariate analysis underwent additional multiple logistic regression analysis. The validity of the multiple logistic regression model was confirmed with the omnibus test and the Hosmer-Lemeshow method. Statistical significance was accepted at *P* < 0.05 with a 95% confidence interval.

## RESULTS

The mean age of the patients in the AF group was 68.1 ± 7.8 years, whereas it was 61.6 ± 9.5 years in the non-AF group. Thus, a significant difference was observed in age between the groups, with the AF group demonstrating a considerably higher mean age (*P* = 0.0001). Furthermore, age was identified as a risk factor for post-CABG NOAF.

Preoperative demographic, clinical, and disease-related parameters that could be associated with NOAF were compared between groups (Table 1).

**Table 1:** Preoperative demographic, patient, and disease-related parameters predisposing to postoperative new-onset atrial fibrillation after coronary artery bypass grafting.

Variables	AF group	Non-AF group	*P*-value
	(n = 115)	(n = 739)	
Age (years)	68.1 ± 7.8	61.6 ± 9.5	0.0001
Female	27 (23.5%)	207 (28%)	0.311
Unstable angina	22 (19.1%)	136 (18.4%)	0.852
History of smoking	47 (41.2%)	378 (51.1%)	0.087
Diabetes mellitus	26 (22.6%)	192 (26%)	0.440
Hypertension	34 (29.6%)	213 (28.8%)	0.870
Obesity (BMI ≥ 30)	18 (15.7%)	121 (16.4%)	0.845
Family history of CAD	33 (28.7%)	209 (28.3%)	0.927
COPD	28 (24.3%)	101 (13.7%)	0.003
Prior myocardial infarction	51 (44.3%)	353 (47.9%)	0.494
Prior PTCA	12 10.4%)	98 (13.3%)	0.400
Renal insufficiency	4 (3.5%)	23 (3.1%)	0.835
Hyperlipidemia	39 (33.8%)	285 (38.5%)	0.447
Prior stroke	3 (2.6%)	8 (1.1%)	0.177
Carotid artery disease	11 (9.6%)	44 (6%)	0.142
Mitral regurgitation, mild/moderate	17 (14.8%)	78 (10.6%)	0.180
Emergency operation	3 (0.4%)	22 (2.6%)	0.827
Low LVEF (≤ 40)	27 (23.5%)	121 (16.4%)	0.061
RCA disease	73 (63.5%)	506 (68.5%)	0.286
LMCA disease	8 (7%)	42 (5.7%)	0.589
EuroSCORE	5.1 ± 3.6	3.9 ± 2.8	0.005
Pulmonary hypertension (≥ 30 mmHg)	78 (67.8%)	413 (56%)	0.017
Pulmonary artery pressure (mean)	33.5 ± 8.7	31.3 ± 8.5	0.009
BSA (m^2^)	1.8 ± 0.1	1.8 ± 0.2	0.319
BMI (kg/m^2^)	26.8 ± 3.3	26.9 ± 3.4	0.668
LVEF (%)	50.8 ± 10.5	50.4 ± 9.4	0.511

Data were expressed as mean ± standard deviation or n (%)

AF=atrial fibrillation; BMI=body mass index; BSA=body surface area; CAD=coronary artery disease; COPD=chronic obstructive pulmonary disease; EuroSCORE=European System for Cardiac Operative Risk Evaluation; LMCA=left main coronary artery; LVEF=left ventricular ejection fraction; PTCA=percutaneous transluminal coronary angioplasty; RCA=right coronary artery

Regarding mean systolic PAP, the AF group presented remarkable elevated values compared to the non-AF group, at 33.5 ± 8.7 mmHg and 31.3 ± 8.5 mmHg, respectively (*P* = 0.017). Similarly, the number of patients with PH (≥ 30 mmHg) was statistically higher in the AF group than in the non-AF group, at 78 (67.8%) and 413 (56%), respectively (*P* = 0.009). The mean European System for Cardiac Operative Risk Evaluation (EuroSCORE) points of the AF group were notably higher than that of the non-AF group (*P* = 0.005), and the number of patients with chronic obstructive pulmonary disease (COPD) was also greater in the AF group.

Perioperative and postoperative parameters of the groups were also compared. The need for inotropes (*P* = 0.033) and the duration of ventilation support (*P* = 0.0001) were significantly higher in the AF group. Additionally, the mean ICU stay was notably longer in the AF group and, as a consequence, the mean hospital stay was also longer in the AF group (Table 2).

**Table 2:** Operative and postoperative parameters of groups.

Variables	AF group	Non-AF group	*P*-value
	(n = 115)	(n = 739)	
LITA usage	104 (90.4%)	676 (91.5%)	0.712
Beating heart	16 (13.9%)	102 (13.8%)	0.974
Mean bypass numbers	2.6 ± 0.8	2.5 ± 0.8	0.384
Need of inotrope	19 (16.5%)	73 (9.9%)	0.033
IABP usage	2 (1.7%)	9 (1.2%)	0.645
Bleeding/reoperation	1 (0.9%)	9 (1.2%)	0.089
Pleural effusion	3 (2.6%)	27 (3.7%)	0.571
Mortality (early)	2 (1.7%)	5 (0.7%)	0.240
Perfusion time (min.)	90.5 ± 21	87.2 ± 23.7	0.366
Cross-clamping time (min.)	75.3 ± 19	74.1 ± 20.9	0.732
ICU stay (days)	3.6 ± 1.6	2.4 ± 1.1	0.0001
Ventilation time (hours)	9.8 ± 12.5	7.1 ± 3.6	0.0001
Hospital stay (days)	7.6 ± 1.9	6.9 ± 1.7	0.001

AF=atrial fibrillation; IABP=intra-aortic balloon pump; ICU=intensive care unit; LITA=left internal thoracic artery

The results of multivariate logistic regression risk analysis for the identification of independent risk factors are shown in Table 3.

**Table 3:** Multivariate logistic regression risk analysis results of groups.

Variables	*P*-value	Odds ratio (95% CI)
Need of inotrope	0.033	1.806 (1.044 - 3.124)
Age (years)	0.0001	1.088 (1.061 - 1.116)
EuroSCORE	0.005	1.152 (1.041 - 1.275)
Ventilation time (hours)	0.0001	1.059 (1.017 - 1.104)
Pulmonary hypertension (≥ 30 mmHg)	0.017	1.659 (1.093 - 2.518)
Pulmonary artery pressure (mean)	0.009	1.027 (1.006 - 1.048)
COPD	0.003	2.033 (1.265 - 3.268)

CI=confidence interval; COPD=chronic obstructive pulmonary disease; EuroSCORE=European System for Cardiac Operative Risk Evaluation

## DISCUSSION

Among patients undergoing CABG, postoperative atrial arrhythmias are prevalent, with AF being the most common and associated with a poor prognosis^[1-3,10]^. However, the etiology of post-CABG NOAF is not yet fully understood and accurately predicting its occurrence remains challenging. Therefore, investigations of the formation mechanism and risk factors are ongoing with the aim of preventing AF.

Among the parameters evaluated in this study, age, COPD, EuroSCORE, PH (≥ 30 mmHg), and mean systolic PAP were identified as risk factors for post-CABG NOAF.

Advanced age is a prominent risk factor for NOAF in patients undergoing CABG as demonstrated by the findings of this investigation, in alignment with the literature to date^[3,9-12]^. Mean age was found to be significantly higher in the AF group of the present study and age was identified as a predictive indicator of post-CABG NOAF (odds ratio [OR]: 1.088). Similarly, in a recent meta-analysis conducted by Alhaddad et al.^[11]^ focusing on AF patients grouped by age of either over or under 80 years, it was found that PH was significantly more prevalent among older patients.

The association between atrial arrhythmias and PH remains the subject of investigations; previous studies have obtained varied results regarding diagnostic outcomes and mortality^[6,7,13-15]^. The present study found no significant difference between groups in term of early mortality within 30 days (AF group, n = 2 [1.7%]; non-AF group, n = 5 [0.7%]; *P* = 0.240).

As the main outcomes of this study, mean systolic PAP and the number of patients with PH (≥ 30 mmHg) were significantly higher in the AF group. Moreover, PH (≥ 30 mmHg) and elevated mean systolic PAP were identified as predictive factors of post-CABG NOAF (OR: 1.659 and OR: 1.027, respectively). For patients with preoperative PH and elevated mean systolic PAP who are undergoing CABG, increased vigilance is required, particularly during the second through fourth postoperative days, when the risk of developing NOAF is the highest. Preventive strategies should accordingly be implemented in these cases, including the potential extension of the ICU monitoring period.

COPD is a well-established parameter and has been reported as an independent risk factor for AF in previous studies^[16-18]^. In the present study, the rate of COPD and EuroSCORE points were significantly higher in the AF group. As in previous studies^[3,8]^, this study has shown that the presence of COPD and high EuroSCORE points are both risk factors and predictive factors for post-CABG NOAF (OR: 2.033 and OR: 1.152, respectively). However, the presence of low left ventricular ejection fraction (≤ 40) was not significantly different between the groups, in contrast to previous reports^[3,8]^.

The relationship between prolonged cross-clamping time and the risk of post-CABG NOAF leads to a variety of outcomes. While some studies have demonstrated a link between aortic cross-clamping duration and post-CABG NOAF^[19-23]^, others have failed to find such an association^[24,25]^. The duration of cross-clamping and perfusion time showed no significant difference between the groups of the present study.

Some researchers also reported that postoperative factors such as the need for mechanical ventilation for > 24 hours and the need for inotropic support were associated with the occurrence of post-CABG NOAF^[23-25]^. These factors were identified as independent predictors of post-CABG NOAF occurrence. Among the parameters considered in the present study, mean duration of ventilation (OR: 1.059) and increased need for inotropic support (OR: 1.806) were identified as independent risk factors and indicators of post-CABG NOAF.

### Limitations

This study has several limitations, particularly including the observational analysis of single-center data and a retrospective design. Patients with a history of arrhythmia were excluded from this study. However, it is possible that patients who had paroxysmal atrial arrhythmia, were asymptomatic, and exhibited normal sinus rhythm on preoperative ECG were overlooked. Additionally, in-hospital patients with brief asymptomatic episodes of AF may have been missed due to the lack of continuous rhythm monitoring after ICU discharge. Another limitation of this retrospective study conducted based on 20 years of data was the lack of detailed echocardiographic findings including information on mitral valve dimensions and function, left/right atrial dilatation, and left ventricular wall thickness/stiffness, which are also important for predicting post-CABG NOAF.

## CONCLUSION

The results of this study suggest that mean systolic PAP and number of patients with PH (≥ 30 mmHg) are significantly higher among patients with AF. Furthermore, PH (≥ 30 mmHg) and elevated mean systolic PAP were identified as independent risk factors and indicators of post-CABG NOAF. To improve the predictive accuracy, additional prospective randomized controlled studies involving larger patient cohorts must be conducted.
